# Gross Hematuria as a Presenting Feature of Posterior Urethral Valves in a Neonate with Normal Antenatal Sonograms

**DOI:** 10.3390/medicines7010005

**Published:** 2020-01-08

**Authors:** Ratna Acharya, Romano DeMarco, Kiran Upadhyay

**Affiliations:** 1 Department of Pediatrics, University of Florida, Gainesville, FL 32610, USA; racharya@ufl.edu; 2 Department of Urology, Division of Pediatric Urology, University of Florida, Gainesville, FL 32610, USA; rdemarco@ufl.edu; 3 Department of Pediatrics, Division of Pediatric Nephrology, University of Florida, Gainesville, FL 32610, USA

**Keywords:** posterior urethral valve, hematuria, renal sonogram

## Abstract

**Background:** Posterior urethral valves (PUVs) are usually suspected during antenatal sonograms or by postnatal evidence of bilateral hydronephrosis with enlarged bladder. Gross hematuria as an initial manifestation of PUV with a history of normal antenatal sonogram is very rare. **Methods:** This is a retrospective chart study. **Results:** We describe a nine-day-old male neonate who presented with gross hematuria and was later found to have a urinary tract infection (UTI) and severe acute kidney injury (AKI). The mother apparently had normal antenatal sonograms with no evidence of fetal hydronephrosis. The child did not have postnatal renal bladder sonogram done until gross hematuria occurred at Day 9 of life. Sonogram showed bilateral severe hydronephrosis and hydroureter with enlarged bladder. The patient underwent ablation of the PUVs after initial bladder decompression with indwelling urethral catheterization. His AKI resolved after prompt treatment of UTI and PUV ablation. **Conclusions:** This report emphasizes the importance of a high index of suspicion for obstructive uropathy in a newborn with gross hematuria irrespective of prenatal sonogram findings.

## 1. Introduction

Congenital abnormalities of the kidneys and urinary tract (CAKUT), including posterior urethral valves (PUVs), are one of the most common causes of chronic kidney disease (CKD) in children. PUVs are a congenital malformation of the posterior urethra and occur exclusively in male infants. The incidence of PUV has remained similar in the past 30 years, about 0.025% among live male births [1,2]. PUVs are the most common cause of bladder outlet obstruction (BOO) in boys that can manifest along a spectrum of severity, ranging from mild obstructive uropathy to CKD and even end stage renal disease (ESRD) [3,4]. With the advancement of the sonogram techniques, antenatal sonograms usually detect most cases of obstructive uropathies; however, some are found only postnatally during routine sonogram, work-up for urinary tract infection (UTI), acute kidney injury (AKI), or other causes. Gross hematuria as an initial manifestation of PUV is very rare. Here, we describe a neonate with apparently normal antenatal sonograms who presented with gross hematuria and was later found to have PUV.

## 2. Materials and Methods

This is a retrospective chart study, and the family of the patient gave the informed consent for inclusion in this study.

## 3. Case Presentation

A nine-day-old African American boy who was born at 36 weeks of gestation by normal vaginal delivery presented with gross blood in the urine for one day. Urine color had been clear until Day 8 of life. Birth weight was 2.7 kg. The mother had an uneventful prenatal course and apparently had a normal prenatal second and early third trimester sonogram (28 weeks) with no fetal hydronephrosis, oligohydramnios, or fetal ascites. The mother did not have a repeat sonogram in the late third trimester. The newborn screen was negative. There was no history of respiratory distress at birth. The patient was discharged home on the second day of life. The patient had been breast-feeding exclusively and making good wet diapers 6-8 times a day. However, he had been fussy for 2–3 days, but had no fever. The parents denied any fall or local genital trauma. Family history was negative for dialysis, transplantation, renal failure, or congenital renal anomalies. His two elder siblings who are five and three years old were apparently healthy.

On physical examination, the vital signs were as follows: temperature, 97.5 °F (36.4 °C), heart rate, 140 beats/min, respiratory rate, 30 breaths/min, oxygen saturation, 99% on room air, and blood pressure, 92/50 mm Hg. The patient was responsive, but appeared fussy. The chest was clear to auscultation bilaterally. Heart sounds were normal with no murmurs. Abdomen examination was significant for bilateral palpable kidneys, but a soft abdomen with a normal appearing uncircumcised male phallus. The right testicle was undescended. There was no peripheral edema, and he was moving all the extremities comfortably.

**Figure 1:**
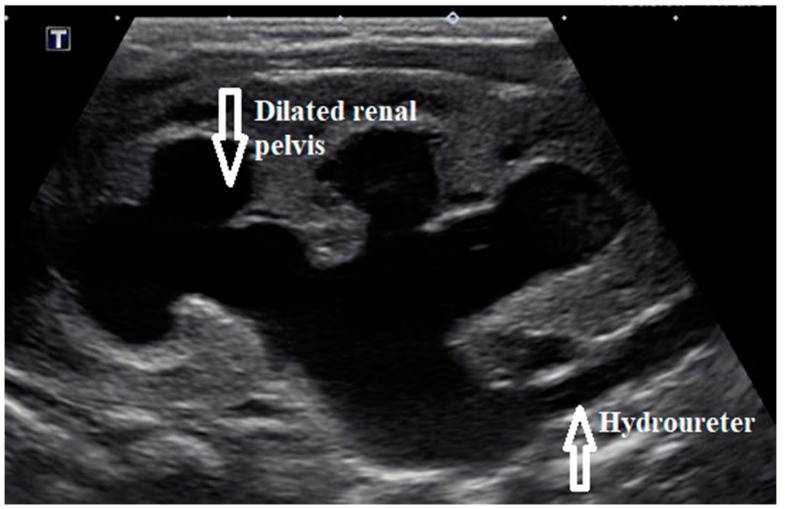
Left renal sonogram showing severe hydronephrosis and hydroureter.

**Figure 2:**
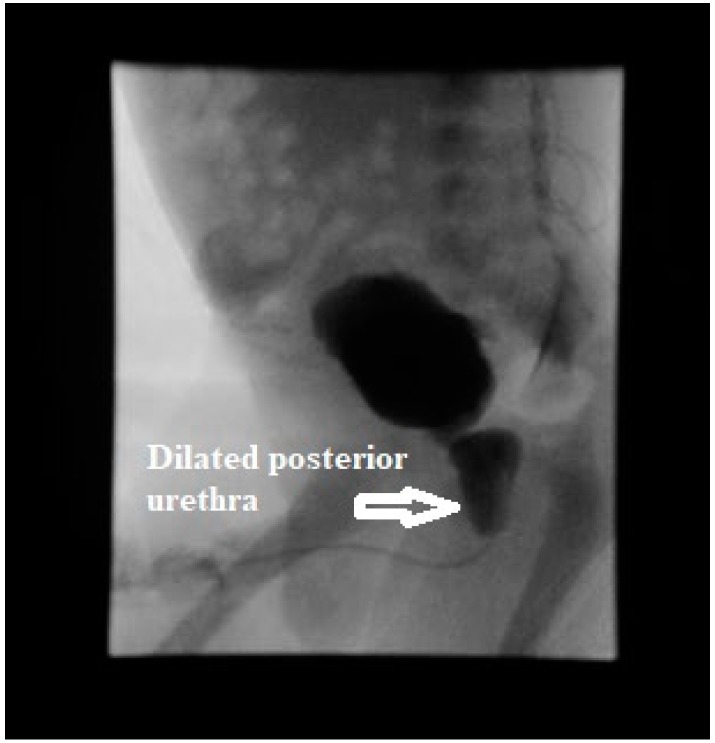
Voiding cystourethrogram showing trabeculated bladder and dilated posterior urethra with no vesicoureteral reflux.

Initial laboratory results showed elevated serum creatinine of 3.5 mg/dL (309.4 µmol/L), severe anion gap metabolic acidosis with bicarbonate of 10 meq/L (10 mmol/L), and potassium of >9 meq/L. (>9 mmol/L). Electrocardiogram showed tall, peaked T waves. Blood counts were normal. Urinalysis showed >100 white blood cells, 2+ protein, large blood with >100 red blood cells, and negative nitrites. Renal bladder sonogram showed bilateral hydronephrosis and hydroureter (Figure 1) along with thick walled bladder with debris and trabeculations. A presumptive diagnosis of lower urinary tract obstruction was made. Voiding cystourethrogram showed a trabeculated bladder and a dilated posterior urethra during the voiding phase consistent with PUV (Figure 2). There was no vesicoureteral reflux (VUR). Initially, bladder was decompressed with an indwelling foley catheter that drained bloody urine. Urine culture grew *Enterobacter* species and was treated with intravenous ampicillin 50 mg/kg/dose twice daily and cefotaxime 50 mg/kg/dose daily (medication dosages adjusted for renal function). Hyperkalemia was managed medically with calcium gluconate, sodium bicarbonate, and insulin glucose along with kayexalate. He underwent cystoscopy, and the valves were ablated the next day; intraoperative finding showed the presence of type I PUV and trabeculated thick walled bladder along with several diverticuli. The UTI in an obstructed bladder probably led to severe AKI in this neonate.

A review of the prenatal sonogram images from the second and early third trimester (28 weeks) showed that the kidneys were not very clearly visualized, and the visualized portion did not have appreciable hydronephrosis. Renal function gradually improved with serum creatinine of 0.9 mg/dL and stable electrolytes at the time of discharge on Day 14 of life. A follow-up renal sonogram obtained a week after PUV ablation showed slight improvement in the bilateral hydronephrosis and hydroureter with persistent echogenic kidneys and loss of corticomedullary differentiation. Follow-up renal sonograms showed stable bilateral hydronephrosis. Three years later, the renal function remained stable with serum creatinine of 0.4–0.6 mg/dL with no recurrence of urinary tract infections and no gross hematuria.

## 4. Discussion

The pathogenesis of PUV appears to be due to an obstructing persistent urogenital membrane. Different types of PUVs have been described, type I being the most common, and is thought to be due to abnormal insertion and absorption of the most distal part of the wolffian ducts during bladder development [5].

PUVs are usually suspected antenatally in about 50% of cases by the sonogram findings of bilateral hydronephrosis. However, the hydronephrosis may not be found in all, even with the high reported sensitivity of the sonogram of about 95% [6]. This may be due to different criteria used to define hydronephrosis, timing of sonogram during pregnancy, and the level of attention to the urinary system by the ultrasonographer [7]. The other half are usually detected in the neonatal period or early infancy during the evaluation of postnatal bilateral hydronephrosis, VUR, UTI, or renal failure. Rarely, some cases present in late childhood or early adolescence with voiding dysfunction and CKD [1]. The radiological manifestation ranges from mild urethral dilatation to bilateral severe hydronephrosis, VUR, urinary ascites, and bladder rupture [8].

UTI is a common cause of gross or microscopic hematuria [9]. In neonates, the clinical manifestations include fussiness, lethargy, feeding intolerance, vomiting, and diarrhea; fever is present in only 20–40% [10]. Obstructed bladder is more prone to UTI due to stagnant urine. UTI in an obstructed bladder requires urgent medical attention, as it can lead to various electrolyte abnormalities and AKI, just as described in this report. To the best of our knowledge, PUV presenting as a delayed onset gross hematuria in a neonate has not been described. There is one case report in the literature about PUV presenting in a one-day-old boy with gross hematuria during his first void, but with normal renal function at presentation [11]. Our patient presented acutely at Day 9 of life with no hematuria in the first few days of life. Our case was also distinct due to the fact that the antenatal sonograms appeared to be normal, but one was not done during the late third trimester. Hence, an apparently normal antenatal sonogram in the early trimesters does not always rule out congenital urinary tract abnormalities. Repeat sonogram performed during the late third trimester is more informative in predicting neonatal CAKUT, which may require surgical intervention [7].

Prenatally, some of the cases of PUVs associated with bilateral severe hydronephrosis and oligohydramnios are managed by the placement of a vesicoamniotic shunt, although the efficacy of this intervention is debated [12]. Postnatally, various measures can be done to relieve the BOO in PUVs. Diversion may include temporary vesicostomy or placement of an indwelling urinary catheter at presentation. However, definite treatment is valve resection/ablation by endoscopy, but some centers may choose to wait until the child is a little older to perform this procedure. Successful decompression of the urinary tract can result in improvement of renal function and/or reversal of obstructive changes. However, significant CKD can occur in patients who had PUV, regardless of whether the valves were ablated or not. One study looked at the long term outcome of 24 patients with a history of PUVs who had ablation of valves before three years of age. At a mean follow-up of 19.5 years, 54% developed CKD, 38% had arterial hypertension, ESRD developed in 20%, and lower urinary tract symptoms were present in 29% [13]. Hence, a long term follow-up of patients with previously treated PUV is mandatory, with a focus on avoiding progressive bladder dysfunction and deterioration of both upper and lower urinary tracts over time.

## 5. Conclusions

Diagnosis of PUV in a neonate might be delayed in circumstances when the antenatal sonogram is reported to be normal and the work-up for UTI and hematuria is limited to urine and blood tests only. As per the American Academy of Pediatrics UTI consensus guideline, any child less than two years of age with first febrile UTI should have a renal bladder sonogram [14]. Since UTI in a male child may coexist with PUV, the sonogram is extremely helpful in identifying obstructive uropathy, irrespective of the antenatal sonogram findings.
